# Thrombolysis of bowel obstruction? A thought-provoking presentation of a common surgical pathology

**DOI:** 10.1093/jscr/rjad189

**Published:** 2023-03-28

**Authors:** Jineel H Raythatha, Bradley Tucker, Geoffrey Lester, Samuel Eather, Ben Lambert

**Affiliations:** Faculty of Medicine and Health, University of Sydney, Sydney, Australia; Mid-North Coast Local Health District, Coffs Harbour, Australia; Faculty of Medicine, University of New South Wales, Sydney, Australia; Faculty of Medicine and Health, University of Sydney, Sydney, Australia; Mid-North Coast Local Health District, Coffs Harbour, Australia; Mid-North Coast Local Health District, Coffs Harbour, Australia; Medical School, University of Notre Dame, Sydney, Australia; Mid-North Coast Local Health District, Coffs Harbour, Australia; Faculty of Medicine, University of New South Wales, Sydney, Australia

## Abstract

We present a unique case of bowel obstruction with a hiatus hernia causing atypical chest pain with dynamic ST-segment elevation in a regional Australian emergency department. The ST elevation only resolved after nasogastric decompression of the bowel obstruction. Early thrombolysis of presumed myocardial infarction led to upper gastrointestinal tract bleeding that could have been avoided with timely diagnosis. An extensive review of literature, in addition to our case report, suggests bowel obstruction is a differential diagnosis for patients who have inferior pattern ST elevation but normal troponin presenting with atypical chest pain, nausea, vomiting and previous abdominal surgery.

## INTRODUCTION

Acute mechanical bowel obstruction is a common surgical emergency, accounting for 20% of emergency surgical operations for abdominal pain [1]. Recognizing and treating these early hold paramount importance due to sequelae such as bowel ischemia and sepsis. We present a very rare and thought-provoking presentation of a common general surgical condition applicable to all surgical trainees and specialists worldwide.

## CASE REPORT

**Figure 1:**
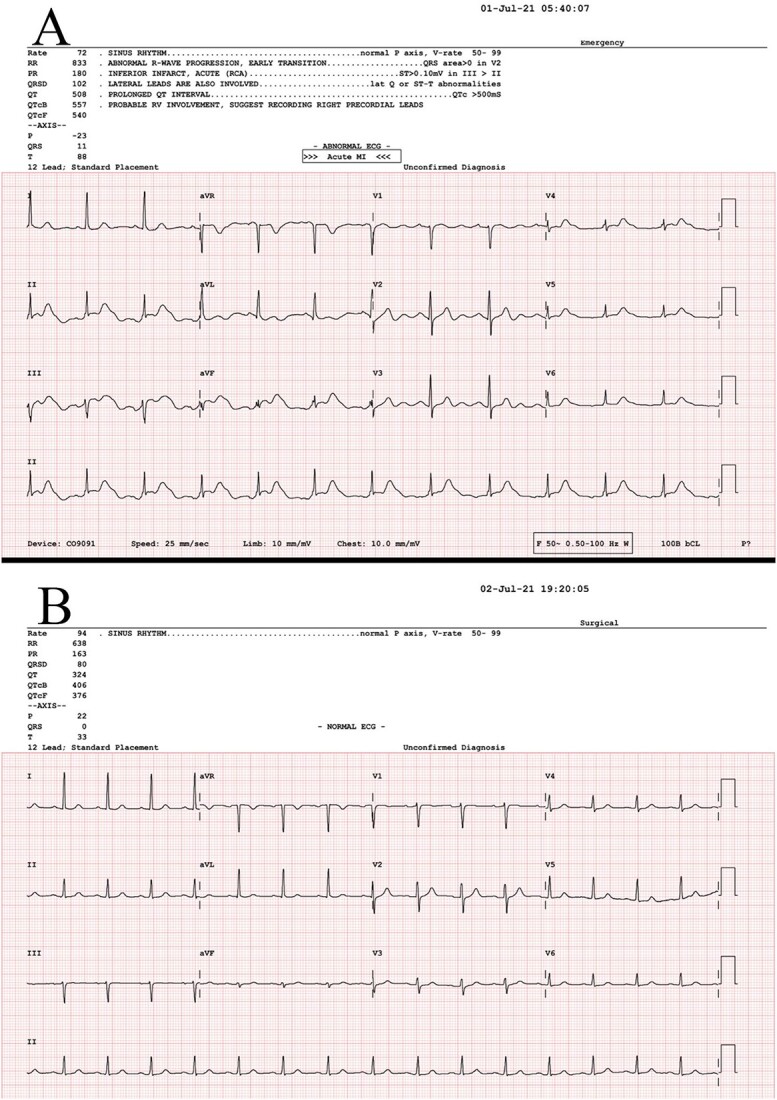
(**A**) ECG at presentation, before thrombolysis, STE in leads II, III and aVF. (**B**) ECG post-NG decompression of distended bowel.

A 78-year-old female presented at 4 am to a regional Australian Hospital with 12 h of severe retrosternal chest pain, associated with diaphoresis, nausea and four episodes of bilious emesis. Her 12-lead electrocardiogram (ECG) met ST-segment elevation myocardial infarction (STEMI) criteria with STE in leads II, III and aVF (Fig. 1A). Her background medical history included a family history of coronary artery disease, dyslipidaemia and peripheral vascular disease, in addition to repaired hiatus hernia, gastro-oesophageal reflux disease and sciatica. She was a lifelong non-smoker and did not engage in heavy alcohol use. Based on the ECG criteria and ongoing chest pain, she was thrombolysed as per local protocols.

**Figure 2:**
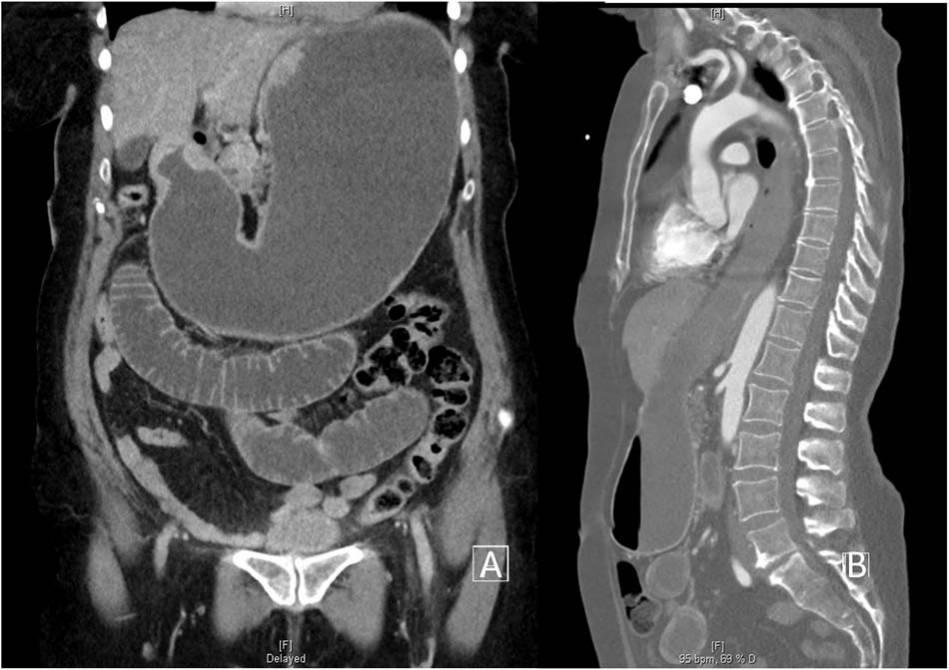
(**A**) Coronal CT cross-section demonstrating gross bowel and stomach distension. (**B**) Sagittal CT cross-section demonstrating obstruction and reflux of stomach contents into oesophagus.

Her initial biochemistry, including high-sensitivity troponin, was within normal limits. A transthoracic echocardiogram showed normal ejection fraction and no segmental wall motion abnormality. She underwent coronary artery catheterization for persistent ST-elevation after thrombolysis, which found only mild non-obstructive coronary artery disease. Due to persisting pain, a computerized tomography (CT) aortogram was performed, demonstrating a significantly dilated stomach, a hiatus hernia and small bowel loops with a transition point suggestive of small bowel obstruction as shown in Fig. 2. Inserting a nasogastric tube for decompression led to significant pain relief and sustained ST-segment elevation (STE) resolution, as shown in Fig. 1B.

Two days later, packed red blood cell transfusions were required for declining haemoglobin to 72 g/L. A CT mesenteric angiogram revealed resolution of the bowel obstruction but active bleeding of a Mallory-Weiss tear in the lower third of the oesophagus, managed with gastroscopy. She recovered well and was discharged home 8 days later. Informed consent to publish form has been obtained and is held by the treating institution as per the local ethics committee guidelines.

## DISCUSSION

Bowel obstruction is one of the most common pathologies in general surgery. Diagnosing and treating this in a timely manner are critical due to consequences such as bowel ischemia, aspiration and sepsis [1]. We present a very rare presentation where bowel obstruction mimics an acute myocardial infarction with ST-segment elevation (STEMI), which itself is a life-threatening medical emergency. Worldwide, our extensive literature review found six published case reports of seven patients describing STE due to distension of the upper gastrointestinal tract [2–7]. There are no reports of complications due to the treatment of bowel obstruction as STEMI. Table 1 shows a summary of these cases. The proposed mechanisms of this symptom and sign mimicry are cardiac displacement due to gastric distension, direct irritative/compressive effect on visceral-cardiac axis [8].

**Table 1:** Review of all case reports of bowel obstruction mimicking ST segment elevation

Case	Age, Sex	Past Medical History	Presentation	Pattern of ST Elevation	Cardiac enzymes	Echo	Angiogram	Treatment
Asada 2006	83F	Subtotal esophagectomy	Worsening chest discomfort	II, III, aVF	Normal	LA, LV compression	Non-obstructive	NG decompression, resolution of STE
Patel 2015	42F	Previous recent SBO	Epigastric pain and dyspnoea	II, III, aVF + V4–V6	Normal	Inferior wall hypokinesis	-	Surgical decompression, resolution of STE
Parikh 2015	86 M	Pancreatitis	Severe epigastric pain	II, III, aVF	Normal	-	Non-obstructive	NG decompression, resolution of STE
Herath 2016	56 M	Nil	Acute vomiting, diaphoresis, abdominal discomfort	II, III, aVF + V1–V3	Normal	Normal	-	NG decompression, resolution of STE
Upadhyay 2017	64 M	Chronic pancreatitis, multiple abdominal surgeries	Abdominal pain, vomiting	II, III, aVF + V5–V6	Normal	Normal	-	Surgical decompression, resolution of STE
Upadhyay 2017	71F	Cholecystectomy, C-section, right-sided femoral hernia	Severe epigastric pain	II, III, aVF + V5–V6	Normal	-	Non-obstructive	Surgical decompression, resolution of STE
Baldwin 2021	50 M	D2 post ventral and hiatal hernia repair, adhesiolysis	Left lower chest pain	II, III, aVF + V5–V6	Normal	Normal	Non-obstructive	NG decompression, resolution of STE

C-section: caesarian section; F: female, LA: left atrium; LV: left Ventricle; M: male; NG: nasogastric; SBO: small bowel obstruction, STE: ST segment elevation.

In each case, including ours, there is atypical chest pain or sometimes abdominal pain accompanied by inferior STE and normal troponin markers. Nearly all patients described as having this phenomena had previous abdominal surgery and hence a risk factor for bowel obstruction with associated symptoms of nausea and vomiting. Variably, inferior wall hypokinesis is demonstrated, likely due to the mechanical restriction of systolic expansion [3]. Cardiac catheterization reveals non-obstructive coronary arteries when performed. In each case, there was the resolution of STE with either nasogastric or surgical decompression.

Unique to our case is the regional Australian location of our hospital. Without immediate access to the cardiac catheterization lab, our patient required thrombolysis of her presumed STEMI rather than a percutaneous coronary intervention. Thrombolysis had several implications in this patient who had a bowel obstruction. About 11.4% of patients after thrombolysis require a transfusion, with the most common site for spontaneous bleeding being gastrointestinal, as observed in our patient who had a Mallory-Weiss tear [9]. Our patient was predisposed to this due to her emesis at presentation and history of hiatus hernia [10]. By increasing the risk of bleeding, thrombolysis also makes laparotomy, if needed, a much riskier operation. Thus, earlier recognition of this phenomenon may have prevented this patient from developing upper GI bleeding. Bedside diagnostic maneuvers that may assist in this are epigastric compression with an ultrasound probe to relieve mechanical compression on the heart while monitoring for transient resolution of STE or repeating an ECG in a standing position [8].

Small bowel obstruction with gastric distension is a very rare but important cause of STE in a patient with prior abdominal surgery presenting with atypical troponin-negative chest pain. Awareness of such cases can lead to earlier delivery of more appropriate care and highlights the primacy of taking a thorough history of presenting illness in presentations of general surgical pathology.

## References

[1] Gore RMMD, Silvers RIMD, Thakrar KHMD, Wenzke DRMD, Mehta UKMD, Newmark GMMD, et al. Bowel obstruction. Radiol Clin North Am 2015;53:1225–40. 26526435 10.1016/j.rcl.2015.06.008

[2] Asada S, Kawasaki T, Taniguchi T, Kamitani T, Kawasaki S, Sugihara H. A case of ST-segment elevation provoked by distended stomach conduit. Int J Cardiol 2006;109:411–3. 15979741 10.1016/j.ijcard.2005.05.036

[3] Baldwin NK, Ives CW, Morgan WS, Bowman MH, Chatterjee A. Small bowel obstruction mimicking acute inferior ST-elevation myocardial infarction. Am J Med 2021;134:599–602. 33316250 10.1016/j.amjmed.2020.11.014

[4] Herath HMMTB, Thushara Matthias A, Keragala BSDP, Udeshika WAE, Kulatunga A. Gastric dilatation and intestinal obstruction mimicking acute coronary syndrome with dynamic electrocardiographic changes. BMC Cardiovasc Disord 2016;16:245. 27899069 10.1186/s12872-016-0423-z PMC5129209

[5] Parikh M, Amor MM, Verma I, Osofsky J, Paladugu M. Small bowel obstruction masquerading as acute ST elevation myocardial infarction. Case Rep Cardiol 2015;2015:1–4. 10.1155/2015/685039 PMC4637458 26587291

[6] Patel K, Chang N-L, Shulik O, DePasquale J, Shamoon F. Small bowel obstruction mimicking acute ST-elevation myocardial infarction. Case Rep Surg 2015;2015:739147–3. 25838963 10.1155/2015/739147 PMC4370232

[7] Upadhyay A, Chauhan S, Jangda U, Bodar V, Al-Chalabi A. Reversible inferolateral ST-segment elevation associated with small bowel obstruction. Case Rep Med 2017;2017:5982910–4. 28465689 10.1155/2017/5982910 PMC5390630

[8] Zhang J, Basrawala H, Patel S, Girn H, Eyvazian V, Wang L, et al. Gastrointestinal distention masquerading as ST-segment elevation myocardial infarction. JACC Case Rep 2020;2:604–10. 34317304 10.1016/j.jaccas.2020.02.016 PMC8298783

[9] Berkowitz S, Granger C, Pieper K, Lee K, Gore J, Simoons M, et al. Incidence and predictors of bleeding after contemporary thrombolytic therapy for myocardial infarction. Circulation (New York, NY) 1997;95:2508–16. 10.1161/01.cir.95.11.2508 9184581

[10] Kortas DY, Haas LS, Simpson WG, Nickl NJ, Gates LK. Mallory-Weiss tear: predisposing factors and predictors of a complicated course. Am J Gastroenterol 2001;96:2863–5. 11693318 10.1111/j.1572-0241.2001.04239.x

